# Neutrophils, Crucial, or Harmful Immune Cells Involved in Coronavirus Infection: A Bioinformatics Study

**DOI:** 10.3389/fgene.2020.00641

**Published:** 2020-06-09

**Authors:** Nima Hemmat, Afshin Derakhshani, Hossein Bannazadeh Baghi, Nicola Silvestris, Behzad Baradaran, Simona De Summa

**Affiliations:** ^1^Immunology Research Center, Tabriz University of Medical Sciences, Tabriz, Iran; ^2^Department of Virology, Faculty of Medicine, Tabriz University of Medical Sciences, Tabriz, Iran; ^3^Medical Oncology Unit, IRCCS IstitutoTumori “Giovanni Paolo II”, Bari, Italy; ^4^Department of Biomedical Sciences and Human Oncology, University of Bari Aldo Moro, Bari, Italy; ^5^Department of Immunology, Faculty of Medicine, Tabriz University of Medical Sciences, Tabriz, Iran; ^6^Molecular Diagnostics and Pharmacogenetics Unit, IRCCS IstitutoTumori “Giovanni Paolo II”, Bari, Italy

**Keywords:** COVID-19, SARS-CoV-2, pneumonia, neutrophil, bioinformatics

## Abstract

The latest member of the *Coronaviridae* family, called SARS-CoV-2, causes the Coronavirus Disease 2019 (COVID-19). The disease has caused a pandemic and is threatening global health. Similar to SARS-CoV, this new virus can potentially infect lower respiratory tract cells and can go on to cause severe acute respiratory tract syndrome, followed by pneumonia and even death in many nations. The molecular mechanism of the disease has not yet been evaluated until now. We analyzed the GSE1739 microarray dataset including 10 SARS-positive PBMC and four normal PBMC. Co-expression network analysis by WGCNA suggested that highly preserved 833 turquoise module with genes were significantly related to SARS-CoV infection. *ELANE, ORM2, RETN, BPI, ARG1, DEFA4, CXCL1, and CAMP* were the most important genes involved in this disease according to GEO2R analysis as well. The GO analysis demonstrated that neutrophil activation and neutrophil degranulation are the most activated biological processes in the SARS infection as well as the neutrophilia, basophilia, and lymphopenia predicted by deconvolution analysis of samples. Thus, using Serpins and Arginase inhibitors during SARS-CoV infection may be beneficial for increasing the survival of SARS-positive patients. Regarding the high similarity of SARS-CoV-2 to SARS-CoV, the use of such inhibitors might be beneficial for COVID-19 patients.

## Introduction

The *Coronaviridae* family, enveloped viruses with a positive-sense single-stranded RNA, can cause numerous diseases, such as respiratory disorders, in both humans and animals (Weiss and Leibowitz, 2011). Among the several types of these viruses, two of them are the most important infectious agents in humans with a high mortality rate. Severe Acute Respiratory Syndrome-related Coronavirus (SARS-CoV) and Middle-East Respiratory Syndrome-related Coronavirus (MERS-CoV) can periodically affect different populations based on regional climatic characteristics as well as genetic diversity (Cui et al., 2019). As a major global concern, the new member of the *Coronaviridae* family, or so-called SARS-CoV-2, is already a pandemic, threatening the global health and causing the Coronavirus Disease 2019 (COVID-19). Similar to SARS-CoV, this new virus can also infect lower respiratory tract cells and can go on to cause severe acute respiratory tract syndrome, followed by pneumonia and even death (Hui et al., 2020). The typical structure of CoV contains 4 structural proteins including spike (S), membrane (M), envelope (E), nucleocapsid (N), and, in some genera, Hemagglutinin esterase (HE) protein (Hussain et al., 2005). These proteins facilitate the life cycle of the virus from attachment to assembly and releasing from the host cell; several non-structural proteins are also involved in preparing and recruiting cellular pathways to achieve a successful viral infection (Fehr and Perlman, 2015).

Given the ability of CoVs to manipulate cellular processes to amplify viral particles, several genes may be transcriptionally altered in the infected-tissues or peripheral blood mononuclear cells (PBMCs) of patients with CoV infection, particularly the genes involved in immune response (Li et al., 2003). It is well-known that the acquired immune system is usually activated to mainly defend against viral infections. However, the important roles of innate immune systems should not be ignored during the invasion of a virus. Neutrophils, as the core cells of innate immunity, contain approximately 60% of the white blood cells within a normal circulation (Petri et al., 2008). Since neutrophils are the first line of defense against the onset of a viral infection, the primary observable phenomenon in the patients is an increase in the number of these kinds of immune cells as well as their elevated lifespans. Despite their ability to protect the host and act as an inhibitory arm against viral infections, neutrophils can also damage host tissues infected with a virus (Galani and Andreakos, 2015). Neutrophils begin the process of defending against microorganisms by releasing antiviral enzymes and toxins stored in their granules. Conversely, the degranulation of these cells in the microenvironment of infected cells could injure the host tissue and deteriorate the disease condition. The neutrophils stimulate the production of pro-inflammatory cytokines such as Tumor necrosis factor-alpha (TNF-alpha) and Interleukin-1 (IL-1), as well as ROS production which, in turn, worsens the disease manifestation (Naumenko et al., 2018). Nowadays, the rapid growth of high-throughput technologies (such as microarray and RNA-seq) has provided new approaches for gene expression profiling. A systems biology approach as a holistic method is a good choice for biomarker discovery, finding novel genes and pathways which are involved in many diseases (Yang et al., 2020).

Therefore, co-expression network reconstruction from the whole transcriptome profile can highlight the critical transcript involved and their relation in a network. There are several co-expression network inference methods t, with weighted gene co-expression network analysis (WGCNA) algorithm the most appropriate to detect key genes related to a sample trait in a network of co-expressed genes (Giulietti et al., 2016). The most interconnected genes in a module, which are defined as hub genes, are often functionally significant and can, therefore, play a main role in many diseases and represent candidate diagnostic biomarkers or potential therapeutic targets. In the current study, we used two system biology approaches, WGCNA and GEO2R as a webtool analyzer to analyze the gene expression of SARS-infected patients, and PBMC to determine the exact molecular mechanisms involved in the pathogenesis of systemic SARS infection and the possible immune responses against to the viruses. Regarding the high similarity of SARS and the Coronavirus disease 2019 (COVID-19) causative agent, SARS-CoV-2, the cellular signaling pathways involved in SARS infection could be the same in SARS-CoV-2 infection. This bioinformatics approach could shed a light on the treatment and prevention of SARS-CoV-2 infection and enhance the knowledge of virus pathogenesis.

## Materials and Methods

### Dataset Selection and Differentially Expressed Genes (DEGs) Identification

NCBI Gene Expression Omnibus (NCBI-GEO) is a simple and free database containing information about the expression of various genes in several types of disorders using the microarray technique. For the current study, we used the GSE1739 dataset (10 SARS-positive PBMC and 4 normal PBMC)which is quantified by the GPL201 platform (Affymetrix Human HG-Focus Target Array). The raw data processed and quantile-normalized with the affy package of R 3.4.1 in Bioconductor. The annotation file published by Affymetrix (Affymetrix Human Genome U133 plus 2.0 Array) was applied to assign probes for appropriate genes. Unconverted data were excluded. Subsequently, the average expression data of identifiers were calculated for each sample. Gene symbols were filtered across all samples using their variance. Finally, the top 5,000 genes with the highest variances were selected for the subsequent analyses. We also used GEO2R as an online tool to find DEGs with *p* < 0.05 and |LogFC| ≥ 2 as the cut-off criteria (Reghunathan et al., 2005).

The control and case groups were assigned to normal PBMC samples and SARS-positive PBMC samples in GEO2R tools. According to the cut-off criteria, 180 genes were detected to be differentially expressed genes (DEGs) in the PBMC of SARS-positive patients compared with normal samples.

### Construction of Co-expression Modules

The gene co-expression network of normal PBMC samples and SARS-positive PBMC samples were reconstructed using WGCNA (Langfelder and Horvath, 2008). Briefly, the matrix of the gene expression profile was converted into the matrix of pairwise gene similarity according to the Pearson test, followed by conversion into the matrix of adjacency. According to already represented scale-free gene co-expression topological algorithm, when the β value is considered as 7, the adjacency matrix met the scale-free topology criteria. For the next step, topological overlap matrix (TOM) and dissimilarity TOM (dissTOM) were created using TOM similarity and dissimilarity modules, based on the correlation of the pairwise gene-co-expression. Finally, the clusters of highly interconnected genes were created with a minimum module size of 30 genes and a cut height of 0.2.

### Construction of Module-Trait Relationships

For determining which modules are strongly related to the SARS disease and calculating this correlation, module eigengene (ME) was recruited to assign expression profiles to each module and the correlation of individual genes was measured using the gene significance (GS). Module membership (MM) was considered as the correlation of the ME and the profile of gene expression for each module. The strong correlation between GS and MM was represented with the closely correlated substantial (central) elements in the modules with SARS (Zhang and Horvath, 2005). Finally, PBMC from SARS-infected people related genes with both GS ≥ 0.8 and MM ≥ 0.9 were selected as hub-genes compared to the control samples. We also evaluated the similarity between DEGs and Hub genes lists using a Venn diagram (https://bioinfogp.cnb.csic.es/tools/venny/).

### Integration of Protein-Protein Interaction (PPI) Network, Pathway Enrichment Analysis, and Modular Analysis

The identified Hub genes and DEGs were subjected to the Search Tool for the Retrieval of Interacting Genes/Proteins (STRING) v11 (Szklarczyk et al., 2019) application of Cytoscape v3.7.2 (Shannon et al., 2003) to find likely PPI with the confidence score ≥ 0.700 and 0 interactors as the cut-off criteria (**Figure 2A**). The DEGs and Hubs with probable interaction were rendered to The Database for Annotation, Visualization, and Integrated Discovery (DAVID) v6.8 and KEGG pathway to find the probable signaling pathways established during the PPI network. The predicted PPI network of Hub genes and DEGs then were analyzed with the Molecular Complex Detection (MDOCE) v1.5.1 to detect highly interconnected regions (clusters) (Bader and Hogue, 2003) with the following cut-off criteria: node degree ≥ 2, node score ≥ 0.2, node density ≥ 0.5, and without haircutting.

### Gene Ontology and Heat Map Analysis

The Hubs and DEGs were subjected to PANTHER classification system to analyze Gene ontology, and biological processes, molecular functions, and cellular components of each gene were distinguished with *p* < 0.05 and gene count ≥ half of the largest number of partnerships in each analysis. Then, the expression value of Hubs in normal samples and SARS-positive samples were put in GraphPad Prism and normalized (smallest value and the largest value of each gene expression were set to 0 and 100%, respectively), to draw a heat map illustrating the pattern of differential gene expression in the samples.

### Candidate Central Nodes and Correlation of Expression

The PPI network of the significant clusters was analyzed with network analyzer of Cytoscape v3.7.2 (Assenov et al., 2008) and 8 genes with the most numbers of interactions were determined as the central nodes including ELANE, ORM2, RETN, BPI, ARG1, DEFA4, CXCL1, and CAMP. Then, the expression correlation of these genes was analyzed with GraphPad Prism and Pearson R test with the cut-off criterion of *p* < 0.05.

### Deconvolution Analysis of PBMC

The normalized expression value of the first 5,000 genes in the PBMC of SARS-positive patients and normal samples were analyzed with the Absolute Immune Signal (ABIS) deconvolution online tool (https://giannimonaco.shinyapps.io/ABIS/) to determine the percentage of each immune cells in the patient and normal samples.

### Statistical Analysis and Graph Making

All the analyses of GEO2R tools, as well as making graphs, were done by GraphPad Prism v8 (GraphPad Software, San Diego, California USA, www.graphpad.com).

## Results

### Finding of WGCNA Modules

**Figure 1:**
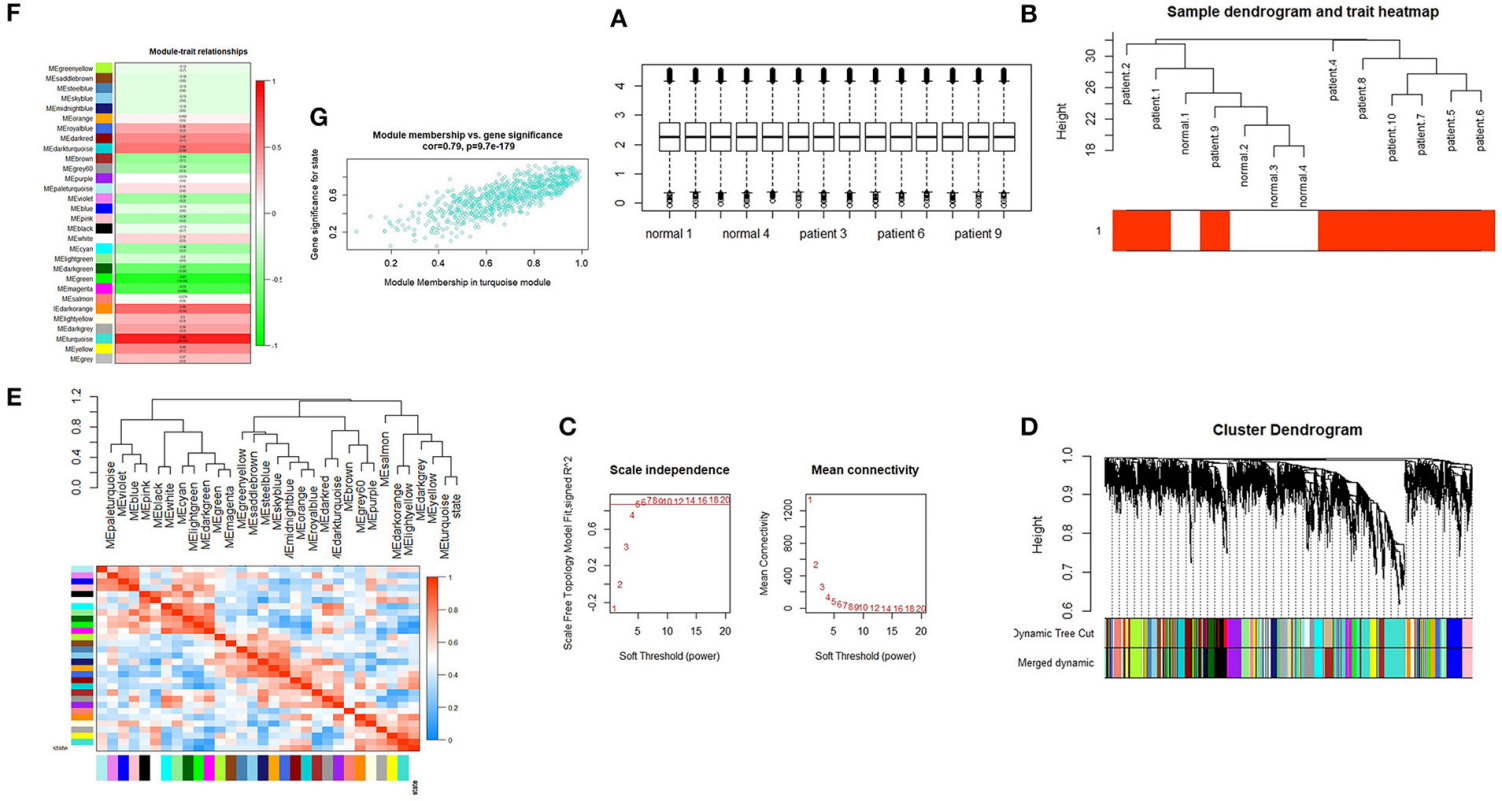
**(A)** Quantile-normalization of samples. Box plots of expression data after normalization. The quantile normalization algorithms were used to adjust the values of the background-subtracted mean pixel intensities of GSE1739. **(B)** Sample clustering to detect outliers. The color is proportional to the pathological stage (Red = SARS infected patient's samples and white = normal samples). **(C)** Selection of the soft-thresholding powers. The left panel shows the scale-free fit index (y-axis) as a function of the soft-thresholding power (x-axis). The right panel displays the mean connectivity (degree, y-axis) as a function of the soft-thresholding power (x-axis). The power was set as 7 for the next analysis. **(D)** Cluster dendrogram and module assignment from WGCNA. The branches correspond to highly interconnected groups of genes. Colors in the horizontal bar represent the modules. **(E)** Module eigengene dendrogram and heatmap. Module-module associations: Eigengene dendrogram and heatmap plot of the adjacencies in the eigengene network including the state which represents the relationships among the modules and the TOF status. **(F)** Module-trait relationship. Each row corresponds to a module eigengene and each column represents one disease status. The numbers in each cell correspond to the corresponding correlation and *p*-values. **(G)** Module features of GS and MM. Module features of GS and MM **(A)** Modules significantly correlated with SARS infected patients and control status (control vs. patient). Each point represents an individual gene within each module, which are plotted by GS on the y-axis and MM on the x-axis.

We performed quantile normalization to reduce the effect of technical noises. The plot of quantiles of expression levels across arrays is shown in Figure 1A. Based on the variance of expression values, a total of 5,000 genes were included in WGCNA. By sample clustering, one sample is excluded in the study (patient 3 as an outlier), thus the rest of the samples were included in the analysis (Figure 1B). For the next step, β threshold power was considered as 7 and a weighted co-expression network was reconstructed for SARS infected patients and normal samples (Figure 1C). The hierarchical clustering dendrogram was represented with 30 modules as illustrated in long branches (Figure 1D-colored bars). The number of genes on each module varied between 2 and 833 (gray and turquoise colors, respectively) (Table S1).

### Module-Trait Association Analysis

Module-module correlation and association of the modules with SARS infected patients were evaluated using eigengenes calculation. The relationships between gene modules are shown in Figure 1E. The turquoise module was positively correlated with this disease (*r* = 0.86, *p* = 2.00E-04) (Table S1). Furthermore, the eigengene network and heatmap indicated a relationship between modules and the SARS infected patients, especially for the turquoise module (Figure 1F).

### DEGs Identification

**Figure 2:**
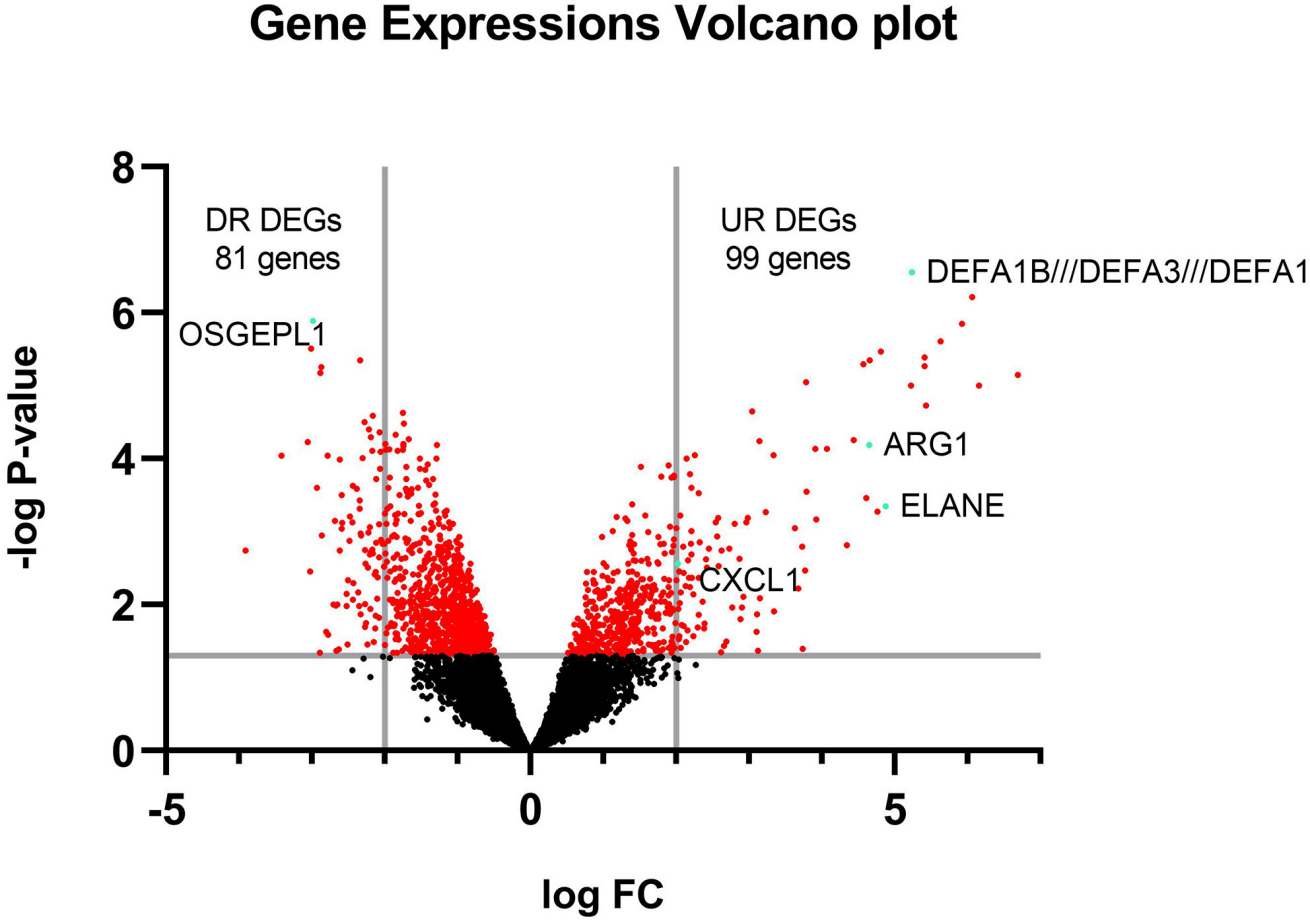
Volcano plot of all gene expressions. The y-axis is separated by a line on -log *P* = 1.301 showing the significance of expression area and the x-axis is separated by 2 lines on logFC = 2 and−2 showing DEGs.

The raw data of the GSE1739 dataset from GEO-NCBI were analyzed with GEO2R tools with *p* < 0.05 and |LogFC| ≥ 2 as the cut-off criteria. 180 genes were detected to be differentially expressed in the PBMC of SARS-positive samples in comparison with normal samples after exerting of cut-off criteria. These genes contained upregulated genes as well as downregulated ones (Figure 2).

### Hub-Genes Detection and Enrichment Analysis

**Figure 3:**
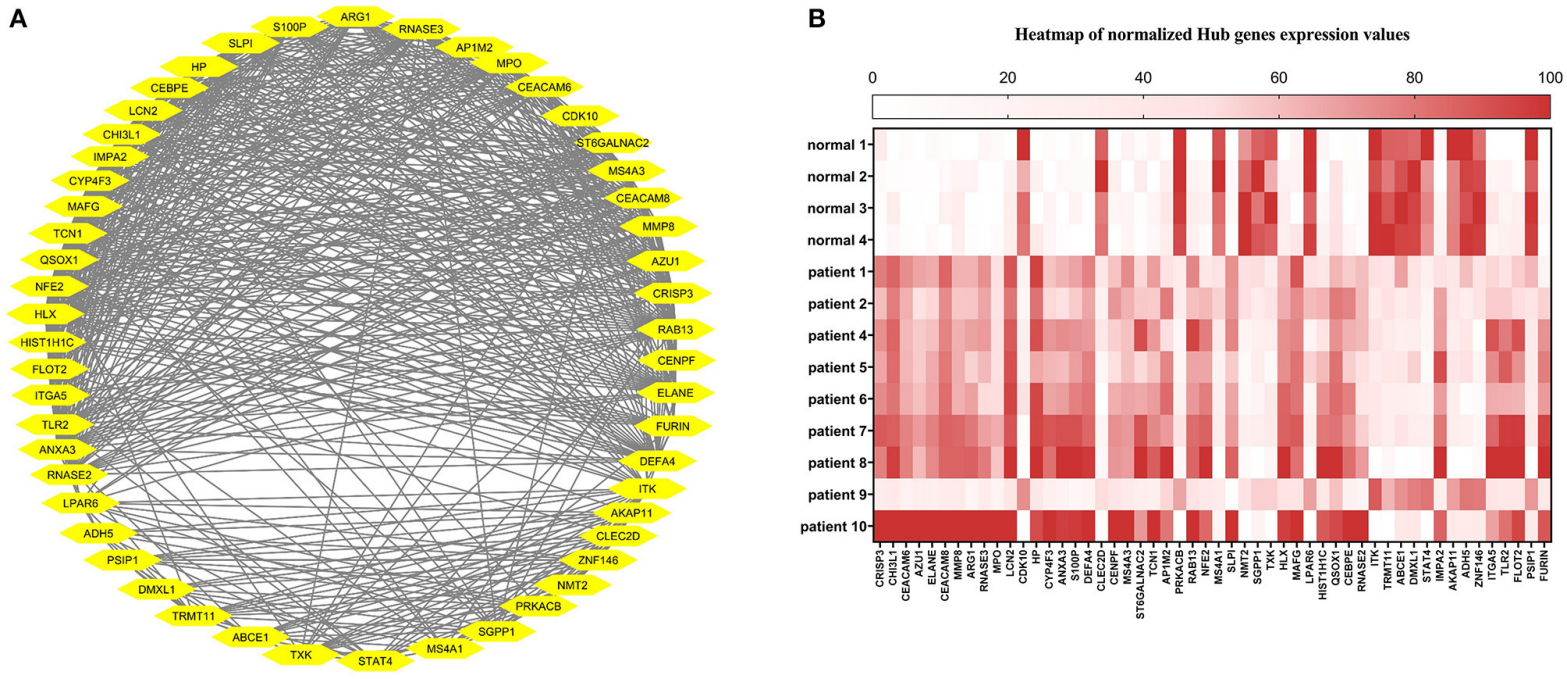
**(A)** Hub gens from the turquoise module. **(B)** Heat map of Hub genes expression in each sample. The expression values from high to low are colored from red to white.

**Figure 4:**
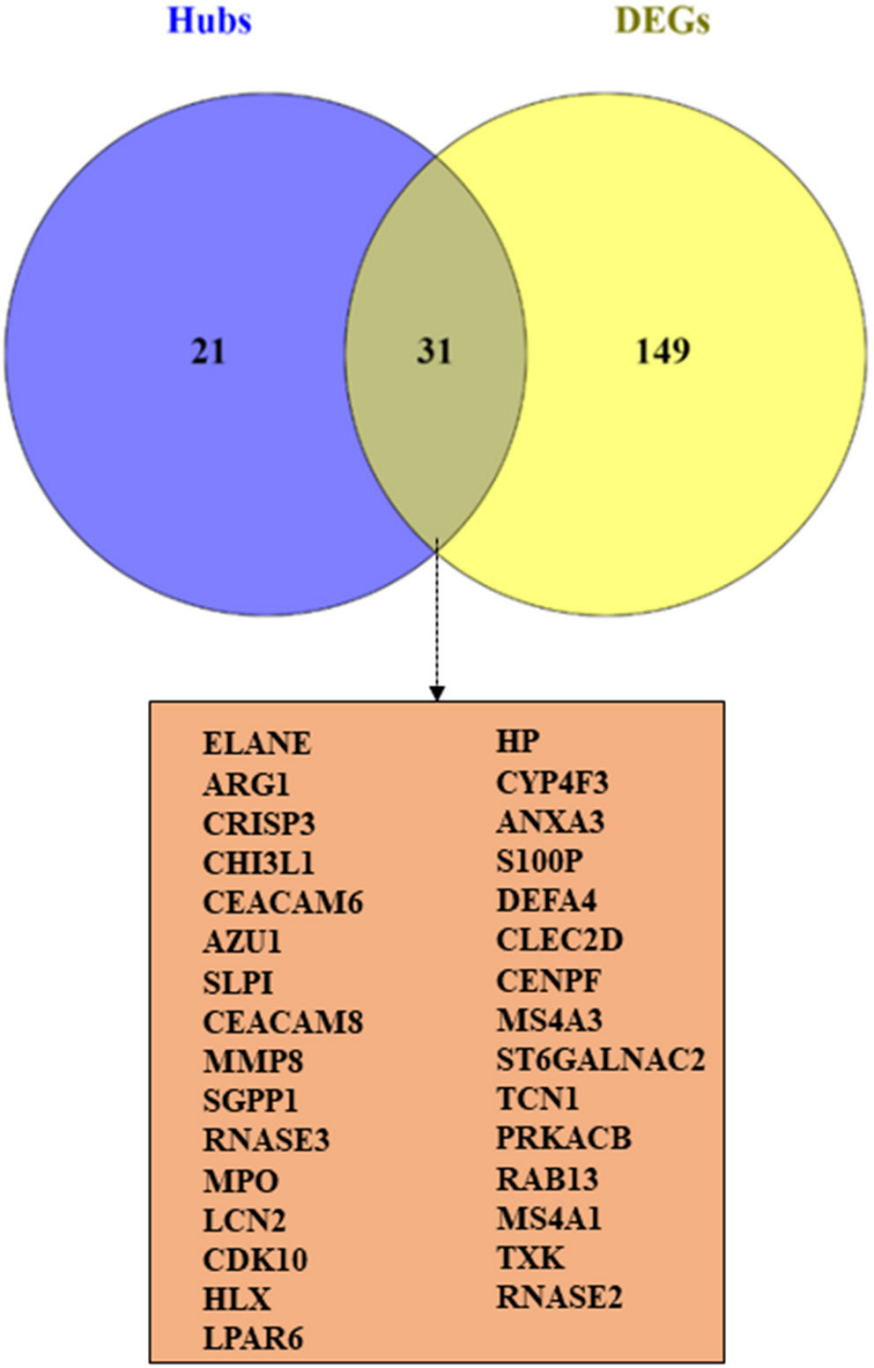
The similarity between DEGs and Hub genes lists using a Venn diagram.

**Figure 5:**
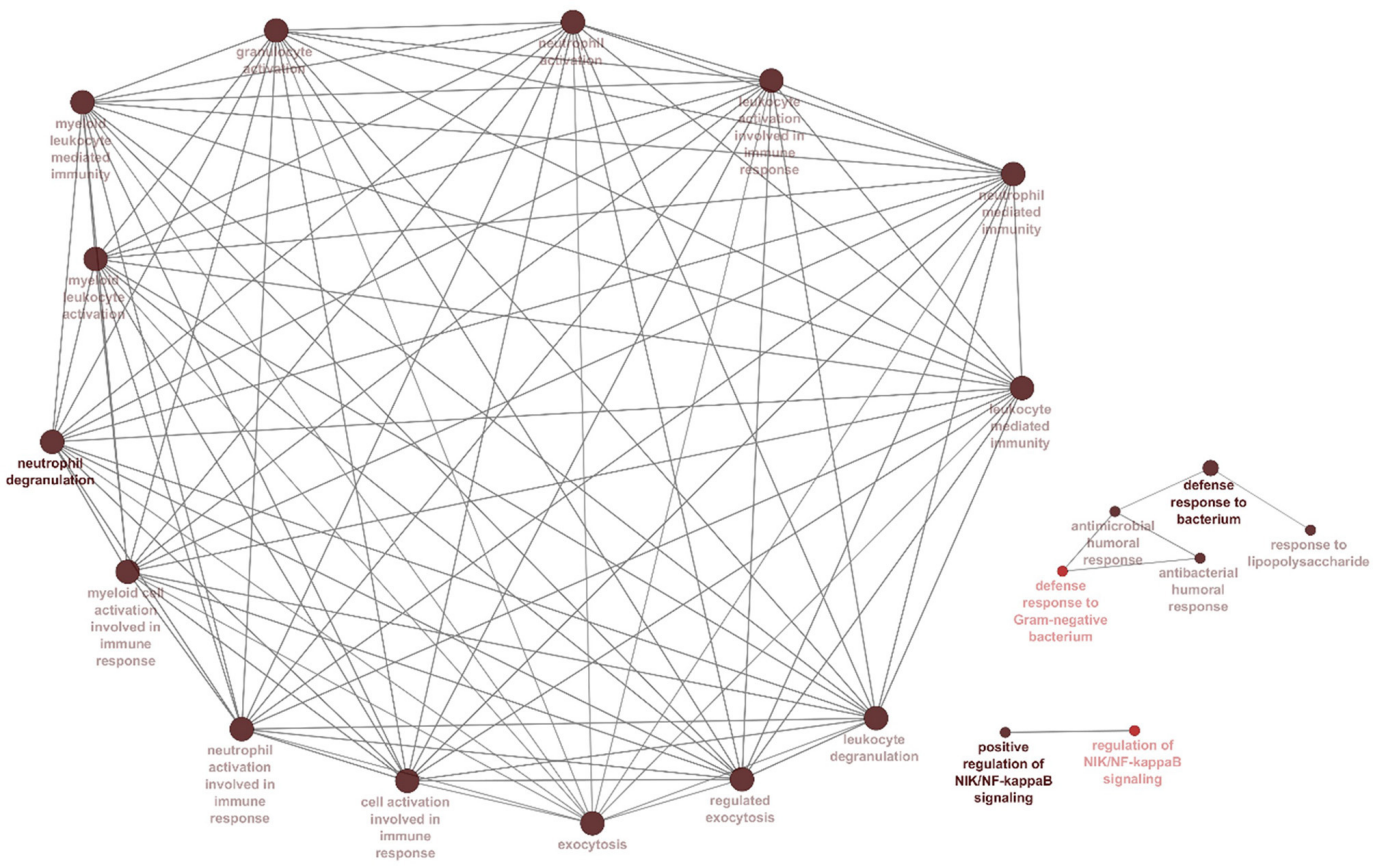
Processes and pathways identified within the hubs and turquoise module. Gene ontology and pathway analysis were performed using significant genes across all datasets. Node size corresponds to the number of associated genes, and node color reflects the statistical significance. The darker pathway nodes, which are more statistically significant, are illustrated with a gradient from red (*p*-value 0.05–0.005) to black (*p* < 0.0005).

The correlation between features (MM and GS) of the turquoise module led to the identification of hub-genes that are highly associated with SARS patient's pathogenesis (Figure 1G). These genes were included as*CRISP3, CHI3L1, CEACAM6, AZU1, ELANE, CEACAM8, MMP8, ARG1, RNASE3, MPO, LCN2, CDK10, HP, CYP4F3, ANXA3, S100P, DEFA4, CLEC2D, CENPF, MS4A3, ST6GALNAC2, TCN1, AP1M2, PRKACB, RAB13, NFE2, MS4A1, SLPI, NMT2, SGPP1, TXK, HLX, MAFG, LPAR6, HIST1H1C, QSOX1, CEBPE, RNASE2, ITK, TRMT11, ABCE1, DMXL1, STAT4, IMPA2, AKAP11, ADH5, ZNF146, ITGA5, TLR2, FLOT2, PSIP1, and FURIN* (Figure 3A). The heatmap of their expression values is also demonstrated in Figure 3B. We showed the similarity between DEGs and Hub genes lists using a Venn diagram (Figure 4). The most considerable pathways which are related to the hub genes were leukocyte mediated immunity, granulocyte activation, neutrophil degranulation, myeloid leukocyte activation, leukocyte mediated immunity, leukocyte degranulation, exocytosis, cell activation involved in immune response, antimicrobial immune defense, and other pathways which presented using the ClueGOtool (Figure 5).

### The Prediction of Likely PPI Network and Module Detection: Roles in the Immune System

**Figure 6:**
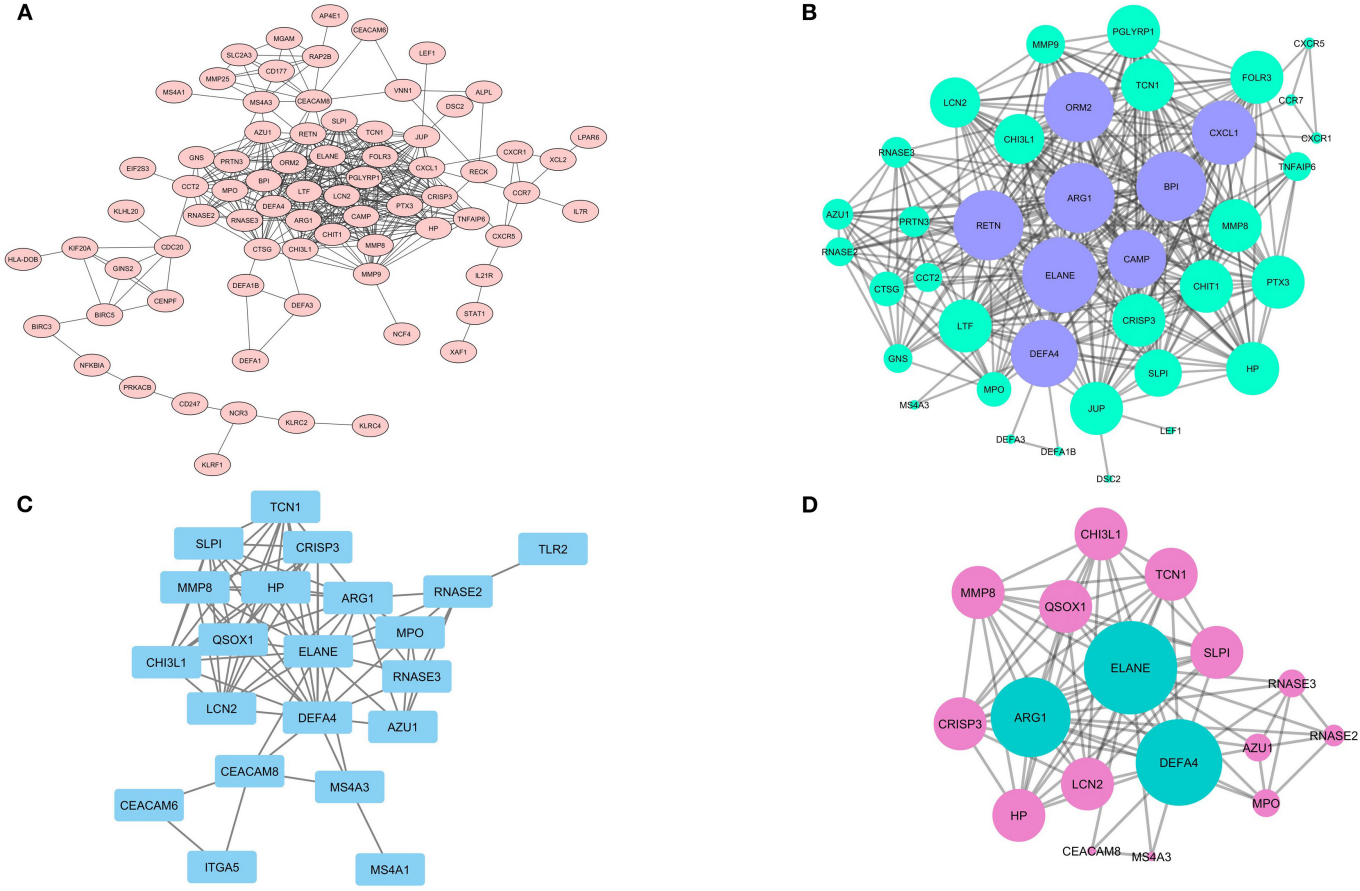
The PPI network of DEGs and analyzed clusters. In the **(A)** network, the possible association between the product of each DEGs is shown with the confidence score ≥ 0.7 and **(B)** network illustrates the likely network between the members of the analyzed cluster by MCODE. **(C)** the network also shows the probable network between Hub genes and network **(D)** prepares a complete association between claustral expression among Hubs.

It was shown that only the production of 75 DEGs (Figure 6A) and 21 Hub genes (Figure 6C) can have interactions in the same network. Eight different clusters were detected in the DEGs PPI network and 2 clusters in Hubs PPI network with the MCODE score of 2.8 to 17.158 and 3.5 to 9.625, respectively. The striking cluster in DEGs PPI network contained 39 nodes, 326 edges, and a MCODE score of 17.158 (Figure 6B) and the remarkable cluster in Hubs PPI network encompassed 17 nodes, 77 edges, and a MCODE score of 9.625 (Figure 6D). DAVID analysis and KEGG pathway showed that the most significant signaling pathway involved in SARS infection is the chemokine signaling pathway (hsa-04062) with *p* = 6.97E-05 (Table 1).

**Table 1:** DAVID and KEGG analysis.

**Signaling pathway**	***p*-value**	**Genes**
hsa04062: Chemokine signaling pathway	6.97E-05	CXCL1, CCR7, CXCR5, CXCR1, NFKBIA, PRKACB, STAT1, XCL2
hsa05200: Pathways in cancer	0.001358243	JUP, LPAR6, MMP9, NFKBIA, LEF1, BIRC5, PRKACB, BIRC3, STAT1
hsa04060: Cytokine-cytokine receptor interaction	0.002237939	CXCL1, CCR7, CXCR5, IL21R, CXCR1, IL7R, XCL2
hsa05140: Leishmaniasis	0.007381227	NCF4, NFKBIA, STAT1, HLA-DOB
hsa04668: TNF signaling pathway	0.02229171	CXCL1, MMP9, NFKBIA, BIRC3
hsa05145: Toxoplasmosis	0.023959103	NFKBIA, BIRC3, STAT1, HLA-DOB
hsa04621: NOD-like receptor signaling pathway	0.039801164	CXCL1, NFKBIA, BIRC3
hsa05161: Hepatitis B	0.048277811	MMP9, NFKBIA, BIRC5, STAT1
hsa05321: Inflammatory bowel disease (IBD)	0.050656748	IL21R, STAT1, HLA-DOB
hsa05412: Arrhythmogenic right ventricular cardiomyopathy (ARVC)	0.054974276	JUP, DSC2, LEF1
hsa05202: Transcriptional misregulation in cancer	0.067983109	JUP, MMP9, ELANE, MPO
hsa04612: Antigen processing and presentation	0.068662464	KLRC4, KLRC2, HLA-DOB

### ELANE Is the Furthermost Important Genes Upregulated During SARS Infection

**Figure 7:**
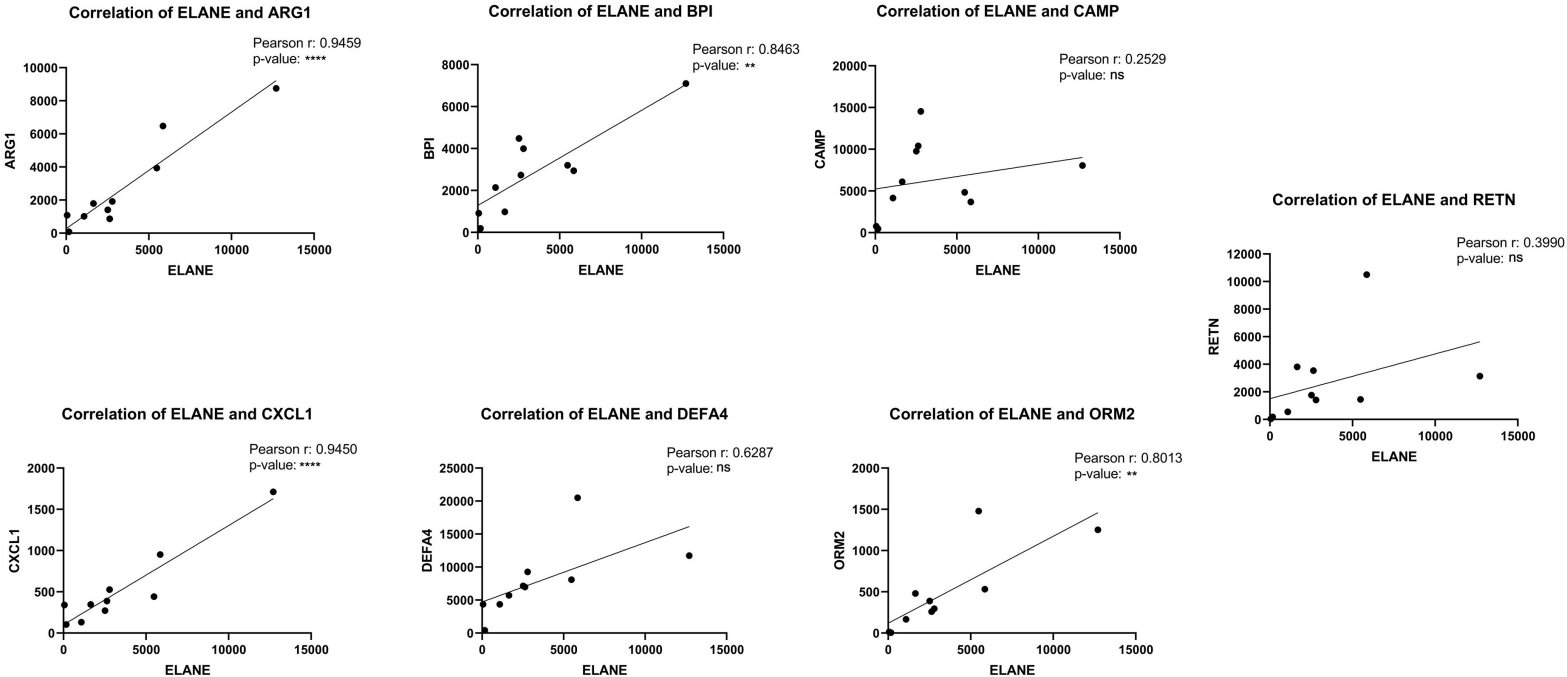
The correlation of expression between ELANE and other central nodes.

The central nodes of the detected cluster were ELANE, ORM2, RETN, BPI, ARG1, DEFA4, CXCL1, and CAMP with the interaction numbers of 30, 28, 28, 28, 28, 27, 26, and 24, respectively (Table S2). ELANE was introduced as the main central nodes of the clusters with a great upregulation level (LogFC = 4.87). The correlations of expression between the other central nodes and ELANE were analyzed with the Pearson R test and the results showed that ELANE has a significant correlation of expression with ARG1, BPI, CXCL1, and ORM2 with Pearson *R* = 0.9459, 0.8463, 0.9450, and 0.8013, respectively. The correlations of ELANE and CAMP, DEFA4, and RETN were not statistically significant (Figure 7).

### Likely Neutrophilia and Lymphopenia During the SARS Infection

Given the use of microarray data from the PBMC samples of SARS-positive and normal samples in this study, the deconvolution analysis was done to detect the percentage of each immune cell in the PBMC samples. The results showed that during SARS-CoV infection, T cells had been significantly decreased in SARS-positive patients compared to normal samples (*p* = 0.0012) as well as the lowered percentage of NK cells (*p* = 0.0331) suggesting probable lymphopenia during SARS. On the other hand, neutrophils and basophils were shown to be significantly increased during this disease with the *p* = 0.0334 and *p* = 0.0018, respectively (Table 2). Together, these data suggest likely lymphopenia and neutrophilia in SARS-positive patients.

**Table 2:** Deconvolution analysis of normal and SARS-positive PBMC samples in the percentage form.

	**N^a^.1**	**N.2**	**N.3**	**N.4**	**P^b^.1**	**P.2**	**P.4**	**P.5**	**P.6**	**P.7**	**P.8**	**P.9**	**P.10**	***p*-value (unpaired *t*-test)**
T Naïve	25.7	8.7	46.1	17	0	2.01	7.02	0.44	0	4.87	0	0	2.05	0.0012 (^**^) ↓
T memory	2.82	29.5	18.1	30.7	23.4	19.6	14.8	16.6	37.7	7.49	10.9	33.3	7.04	0.8537 (ns^c^)
B Naïve	2.68	13	10.1	10.1	9.85	6.02	0	6.11	4.06	3.16	2.29	4.94	7.59	0.0714 (ns)
B memory	2.44	1.97	0.53	1.07	0	1.33	2.7	0	1.82	0	1.39	0.9	0	0.3171 (ns)
Plasmablasts	0.44	1.27	0.68	0.38	1.85	1.07	0.37	0.67	1.15	1.94	0.76	0.76	2.84	0.2033 (ns)
NK^d^	62.9	45.1	26	31.2	20.2	16.2	6.66	6.9	4.06	7.54	3.02	62.5	3.83	0.0331 (^*^)↓
pDCs^e^	2.1	0.47	6.33	3.53	9.71	4.54	5.2	5.11	2.09	6.75	2.15	0.9	4.48	0.3813 (ns)
Neutrophils	1.96	2.59	4.19	2.5	6.23	22	42.3	14.8	21.8	45.7	67.2	2.95	55.2	0.0334 (^*^)↑
Basophils	2.68	1.45	3.01	2.25	17.1	16.9	8.37	13.9	17.5	8.16	11	5.58	8.73	0.0018 (^**^)↑
mDCs^f^	0	0.02	0.03	0.06	0.09	0.21	0	0.44	0.23	0.07	0.18	0.05	0.14	0.0824 (ns)
Monocytes	22.2	30.3	34.8	35.2	30.6	25	34.6	34.7	15.6	12.8	9.39	26.7	12.6	0.1617 (ns)

a *, Normal*;

b *, Patient*;

c *, not significant*;

d *, Natural killer*;

e *, Plasmacytoid dendritic cells*;

f *, Myeloid dendritic cells*.

** *p < 0.01*;

* *p < 0.05*;

↑, *increase;* ↓, *decrease*.

### ELANE Expression Is a Promising Marker for the Number of Neutrophils in SARS Patients

**Figure 8:**
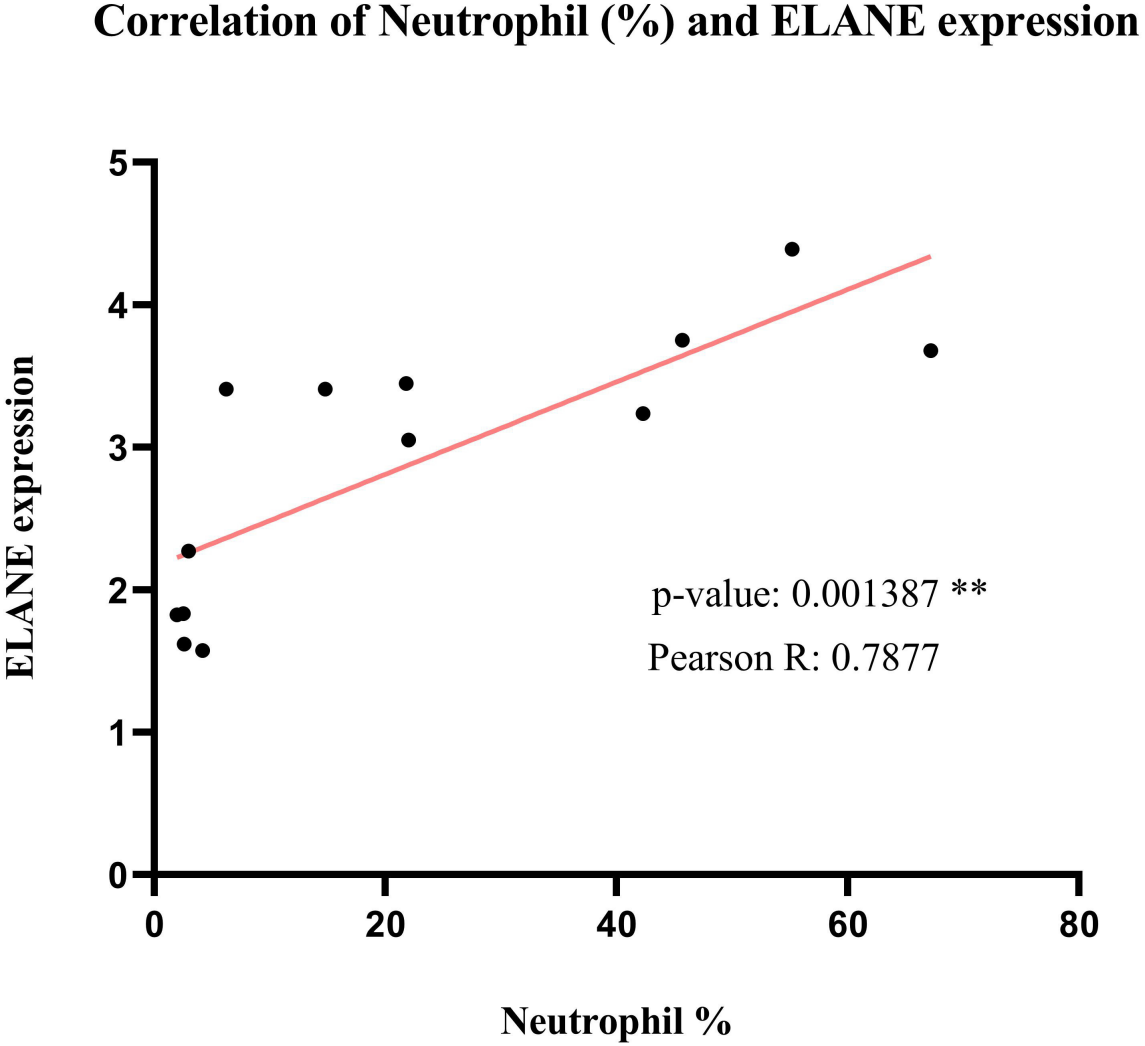
The correlation analysis of Neutrophil percentage and normalized ELANE expression values in normal samples and SARS-positive samples. The result shows that there is a significant positive correlation between these 2 parameters.

As predicted before, ELANE is the main central node differentially expressed during SARS compared to healthy persons. To find the correlation of ELANE expression and percentage of neutrophil, the expression value of ELANE in each sample was compared with the percentage of neutrophil in the same sample. The analysis demonstrated that there is a significant correlation (*p* = 0.001387 and Pearson *R* = 0.7877) between the expression value of ELANE and the number of neutrophils, suggesting that any amount of ELANE upregulation could increase the number of neutrophils in the PBMC of SARS patients in an ELANE expression-dependent manner (Figure 8).

### Activation of Immune Cells in SARS Infection

**Figure 9:**
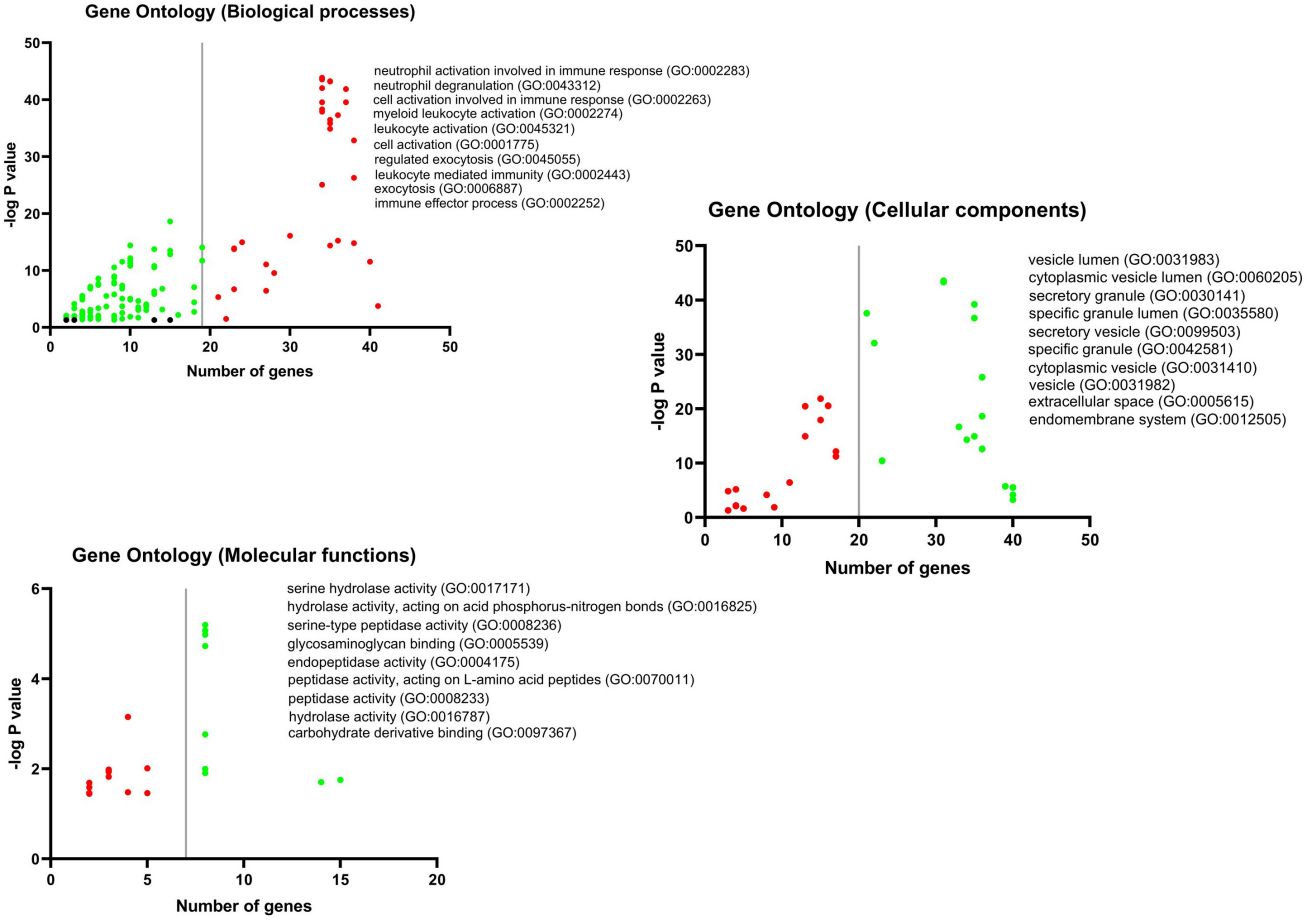
GO analysis of DEGs and Hubs. The graphs show the possible biological process, cellular components, and molecular functions that all DEGs and Hubs could participate in.

GO analysis DEGs and Hubs together demonstrated that neutrophil activation (GO: 0002283) and neutrophil degranulation (GO: 0043312) are the most activated biological processes in a SARS infection. Moreover, the striking molecular functions during SARS infection was predicted to be serine hydrolase activity (GO: 0017171), hydrolase activity on acid phosphorus-nitrogen bonds (GO: 0016825), and serine-type peptidase activity (GO: 0008236). The DEGs and Hubs were also predicted to participate in the following cellular components: vesicle lumen (GO: 0031983), cytoplasmic vesicle lumen (GO: 0060205), and secretory granule (GO: 0030141) (Figure 9).

## Discussion

As SARS-CoV-2 threatens world health, it is essential to find the molecular mechanisms involved during this infection to find the proper treatment and prevention approaches. Given the similarity of SARS-CoV-2 to another member of the *Coronaviridae* family, SARS-CoV, we analyzed the expression profile of SARS-positive patients compared to normal samples to find the molecular mechanisms involved in this infection by using WGCNA and the other systems biology approaches. In the present study, we analyzed the GSE1739 dataset. Co-expression network analysis by the WGCNA suggested that a highly preserved turquoise module with 833 genes was significantly correlated to the SARS-positive patients. Toll-like receptors (TLRs), which are known as the initiators of innate immune response, and neutrophils, which restrict viral replication, can also trigger the overproduction of cytokines or cause a so-called “cytokine storm” which aggravates the virus-caused disease (Shirey et al., 2013). The production of TLR-3 has been also reported in the SARS-CoV infection which, despite its ability to protect host cells against the virus, might be responsible for lung tissue injury (Totura et al., 2015). Regarding the GO analysis, neutrophil activation and neutrophil degranulation were the most activated biological processes during SARS-CoV infection in the current study. Besides, the deconvolution analysis of patients' PBMC compared to the normal samples showed a significant increase in the percentage of neutrophils and basophils as well as a decreased level of T cells suggesting the probable neutrophilia, basophilia, and lymphopenia during SARS pathogenesis. It has been demonstrated that neutrophilia is one of the most dangerous clinical manifestations in patients with SARS (Tsui et al., 2003). Furthermore, neutrophilia has been reported to be associated with the induction of hemorrhagic lesions in the lungs of rats with coronavirus infection as well as its ability to worsen the clinical conditions by creating an inflammatory response (Haick et al., 2014). Huang et al. reported that elevated levels of pro-inflammatory cytokines in COVID-19 patients create the cytokine storm condition previously seen in SARS patients, increasing the lung tissue injury and deteriorating the fatality rate of SARS-CoV-2 infection. Moreover, they showed that in COVID-19 patients' sera with the need for intensive care unit (ICU)admission, there was a high concentration of Granulocyte-colony stimulating factor (G-CSF), C-X-C Motif Chemokine Ligand 10 (CXCL10), Monocyte chemoattractant protein 1 (MCP1), Macrophage inflammatory protein 1 A (MIP1A), and TNF-α. These cytokines are responsible for attracting neutrophils to the inflammatory sites (Huang et al., 2020). The above data could highlight the role of neutrophil in the induction of illness severity in COVID-19 patients.

The striking upregulation of ELANE (a neutrophil elastase) detected in the current study, as well as the significant positive correlation between the ELANE expression and neutrophil percentage (analyzed by ABIS), also underlines the induction of likely ELANE-dependent neutrophilia in the PBMC of SARS-positive patients. ELANE is a kind of serine proteinase secreted by PBMCs as well as neutrophils through an inflammatory response and could give rise to the maturation of neutrophils and consequent neutrophilia (Belaaouaj et al., 2000). Regarding the effects of neutrophilia on the induction of hemorrhagic lesions in the lungs of coronavirus-positive bodies and overexpression of ELANE in patients with SARS, as an important factor for causing neutrophilia, it could be speculated that using ELANE inhibitors, such as Serpins (Serine Protease Inhibitors), might ameliorate the hemorrhaging of the lungs in SARS patients. CT scan reports have shown a great number of COVID-19 patients with pneumonia followed by a pulmonary hemorrhage, as one of the leading causes of death in COVID-19 patients (Shi et al., 2020). According to the high similarity between SARS-CoV-2 and SARS-CoV, ELANE expression-dependent neutrophilia might be the reason for pulmonary hemorrhages in COVID-19 patients. The use of Serine protease inhibitors (Serpins) could be helpful to decrease the mortality rate. As shown above, ARG1 (arginase 1) is another upregulated gene with a significant correlation of expression with ELANE in SARS-positive patients. This enzyme is also reported to be involved in the inflammation of the lungs (Cloots et al., 2017), and it could be hypothesized that the correlated expression of ELANE and ARG1 might deteriorate the hemorrhaging of the lungs in SARS-positive patients and, similarly, COVID-19 patients.

Prescription of arginase inhibitors together with Serpins in COVID-19 patients could be considered as a therapeutic approach. The correlation of expression analysis in this study also demonstrated the correlation of ELANE and CXCL1 (chemokine C-X-C ligand 1). CXCL1 can be expressed by neutrophils, as well as macrophages and epithelial cells, and chemically attract neutrophils to the inflammation sites (Iida and Grotendorst, 1990). The observed overexpression of this chemokine analyzed before might increase the number of neutrophils in the lung epithelial and consequent neutrophilia in this tissue.

Overall, it is hypothesized that neutrophils are the most vital immune cells involved in SARS-CoV infection, increasing the inflammation and hemorrhagic lesions in the lungs of infected patients. ELANE, ARG1, and CXCL1 enhance the neutrophilia condition in these patients by their overexpression. Thus, using Serpins and Arginase inhibitors during SARS-CoV infection can help the survival of SARS-positive patients. Regarding the high similarity of SARS-CoV-2 to SARS-CoV, the use of such inhibitors might be beneficial for COVID-19 patients. However, the exact expression of ELANE, ARG1, and CXCL1, as well as neutrophil counting in COVID-19 patients, should be investigated by further studies to shed further light on COVID-19 treatment.

## Data Availability Statement

Publicly available datasets were analyzed in this study. This data can be found here: **GSE1739(GEO)**.

## Author Contributions

NH, AD, and BB: designed the study. AD and HB: performed bioinformatic analyses. NS and SD: revised methodologies and the manuscript. All authors contributed to the article and approved the submitted version.

## Conflict of Interest

The authors declare that the research was conducted in the absence of any commercial or financial relationships that could be construed as a potential conflict of interest.
