# Supplementary material for: Neutrophils, Crucial, or Harmful Immune Cells Involved in Coronavirus Infection: A Bioinformatics Study
Source: Front Genet. 2020 Jun 9;11:641. doi: 10.3389/fgene.2020.00641 (PMC7296827; doi:10.3389/fgene.2020.00641)
Supplement: Supplementary file 1 [file Data_Sheet_1.docx]

**Supplementary files**

**Table 1:Module color characterization. The co-expression modules were identified by WGCNA**.

| Number | Module Color | Correlation | *p*-value | #Genes |
| --- | --- | --- | --- | --- |
| 1 | Green | -0.87 | 1.00E-04 | 231 |
| 2 | Turquoise | 0.86 | 2.00E-04 | 833 |
| 3 | Magenta | -0.72 | 0.006 | 173 |
| 4 | Darkgreen | -0.57 | 0.04 | 97 |
| 5 | Darkorange | 0.58 | 0.04 | 85 |
| 6 | Darkturquoise | 0.54 | 0.06 | 97 |
| 7 | Brown | -0.44 | 0.1 | 412 |
| 8 | Darkred | 0.48 | 0.1 | 102 |
| 9 | Yellow | 0.45 | 0.1 | 258 |
| 10 | Cyan | -0.38 | 0.2 | 121 |
| 11 | Darkgrey | 0.39 | 0.2 | 95 |
| 12 | Pink | -0.35 | 0.2 | 175 |
| 13 | Royalblue | 0.36 | 0.2 | 104 |
| 14 | Violet | -0.39 | 0.2 | 43 |
| 15 | Grey60 | -0.34 | 0.3 | 234 |
| 16 | Lightyellow | 0.3 | 0.3 | 108 |
| 17 | Grey | 0.27 | 0.4 | 2 |
| 18 | Lightgreen | -0.2 | 0.5 | 114 |
| 19 | White | 0.19 | 0.5 | 79 |
| 20 | Blue | -0.14 | 0.6 | 273 |
| 21 | Midnightblue | -0.18 | 0.6 | 121 |
| 22 | Paleturquoise | 0.15 | 0.6 | 58 |
| 23 | Saddlebrown | -0.16 | 0.6 | 69 |
| 24 | Skyblue | -0.15 | 0.6 | 79 |
| 25 | Steelblue | -0.15 | 0.6 | 69 |
| 26 | Black | -0.13 | 0.7 | 421 |
| 27 | Greenyellow | -0.12 | 0.7 | 166 |
| 28 | Orange | 0.092 | 0.8 | 86 |
| 29 | Salmon | -0.079 | 0.8 | 128 |
| 30 | Purple | 0.019 | 0.9 | 167 |

**Table 2: The DEGs cluster members expression and interaction**

| Genes | Number of interactions | LogFC |
| --- | --- | --- |
| ELANE | 30 | 4.875981 |
| ORM2 | 28 | 3.3459616 |
| RETN | 28 | 2.6546512 |
| BPI | 28 | 5.6329126 |
| ARG1 | 28 | 4.6493704 |
| DEFA4 | 27 | 4.808832 |
| CXCL1 | 26 | 2.024911 |
| CAMP | 24 | 5.2252987 |
| CRISP3 | 22 | 6.1599359 |
| CHIT1 | 22 | 2.2966493 |
| PGLYRP1 | 22 | 4.7658212 |
| TCN1 | 22 | 3.3396451 |
| LTF | 22 | 6.6895496 |
| HP | 22 | 4.6576865 |
| JUP | 22 | -2.1919977 |
| MMP8 | 22 | 3.7697814 |
| FOLR3 | 22 | 2.6910585 |
| PTX3 | 22 | 2.4491357 |
| LCN2 | 21 | 5.4127917 |
| CHI3L1 | 21 | 5.9222558 |
| SLPI | 20 | 2.5783832 |
| MMP9 | 17 | 3.9218383 |
| MPO | 15 | 3.7909637 |
| CTSG | 15 | 2.7269637 |
| PRTN3 | 13 | 3.1115492 |
| RNASE3 | 13 | 4.5669813 |
| AZU1 | 13 | 5.4111128 |
| TNFAIP6 | 12 | 2.8717674 |
| RNASE2 | 12 | 2.2583598 |
| GNS | 12 | -2.78274 |
| CCT2 | 12 | -2.3430773 |
| CXCR1 | 3 | 2.9632704 |
| CXCR5 | 3 | -2.3014234 |
| CCR7 | 3 | -2.0062062 |
| MS4A3 | 2 | 3.7826754 |
| DEFA1B | 2 | 5.237359 |
| DEFA3 | 2 | 5.237359 |
| DSC2 | 1 | 2.3070142 |
| LEF1 | 1 | -2.0788131 |
